# Community Resilience and Long-Term Impacts of Mental Health and Psychosocial Support in Northern Rwanda

**DOI:** 10.3390/medsci6040094

**Published:** 2018-10-24

**Authors:** Yuko Otake

**Affiliations:** 1 School of Anthropology & Museum Ethnography, University of Oxford, Oxford OX2 6PE, UK; Yuko.otake@anthro.ox.ac.uk; 2 Japan Society for the Promotion of Science, 5-3-1 Kojimachi, Chiyoda-ku, Tokyo 102-0083, Japan

**Keywords:** Rwanda, resilience, wellbeing, reconciliation, mental health and psychosocial support

## Abstract

Recently, discussions have considered how mental health and psychosocial support (MHPSS) can build upon local resilience in war-affected settings. To contribute to the knowledge in this field, the paper explored the gap between MHPSS and local communities in terms of perceived mental health problems and healing processes, and how the gap could be filled. Qualitative research was conducted in northern Rwanda with 43 participants between 2015 and 2016. Findings revealed how three particular gaps can isolate MHPSS recipients in their local community. First, whereas MHPSS applies bio-psychological frameworks to post-genocide mental health, community conceptualisations emphasise social aspects of suffering. Second, unlike MHPSS which encourages ‘talking’ about trauma, ‘practicing’ mutual support plays a major role in the community healing process. Third, MHPSS focuses on one part of the community (those who share the same background) and facilitates their healing in intervention groups. However, healing in natural communities continues in everyday life, through mutual support among different people. Despite these gaps, MHPSS recipients can be (re)integrated into the community through sharing suffering narratives and sharing life with other community members. The paper highlights the ways in which MHPSS could inclusively support different social groups in the overall geographical community, allowing members to preserve the existing reciprocity and recover collective life through their own initiatives.

## 1. Introduction

The United Nations Inter-Agency Standing Committee (IASC) Guidelines define ‘mental health and psychosocial support (MHPSS)’ as multi-layered support to address psychological-nature problems (e.g., grief, severe mental disorder, depression, anxiety, post-traumatic stress disorder (PTSD)) and social-nature problems (e.g., extreme poverty, political oppression, family separation, community destruction) [1]. Although the MHPSS provision has become increasingly popular across different emergency settings, its long-term consequences are unknown. This paper presents the case of Rwanda, where a gap has arisen between MHPSS recipients and others in local communities over 20 years of support provision, and considers how MHPSS provision could be improved in war-affected settings.

Studies of MHPSS in war-affected populations had emerged before the IASC guidelines were established, resulting in the proposal of three major approaches: biomedical, cultural/anthropological, and psychosocial models [2]. The biomedical approach has universally applied Western psychiatric diagnoses such as PTSD [3,4,5] and psychotherapeutic techniques such as individual talking therapy, cognitive-behavioural therapy (CBT), and narrative exposure therapy (NET) in war-affected populations [6,7,8]. However, this universal application of the Western biomedical model has been criticised by cultural/anthropological studies for imposing Western biomedical perspectives and failing to adequately meet local needs [9,10,11]. One representative exemplar of these unmet needs is UNICEF’s (United Nations Children’s Fund) trauma recovery programme in post-genocide Rwanda. This provided psychological counselling for traumatised children; however, less than 1% of the target population used the counselling service [12]. This so-called ‘Rwanda experience’ revealed a serious gap between biomedical MHPSS programmes and local needs and reality. Mental health and psychosocial support programmes are unlikely to be successful when local perceptions of suffering and healing are not sufficiently respected based on culture and society [13].

In response to such a fruitless experience, through the biomedical approach an advanced version of the biomedical model was developed: the culturally-adapted biomedical model [14,15]. Researchers using this model investigated local perceptions of war-related syndromes and tailored assessment tools for local use, rather than directly applying Western diagnostic criteria [16,17,18]. They then conducted local adaptations of psychotherapy and evaluated its effects on local syndromes using tailored instruments in randomised-controlled trials [19,20]. The cultural adaptation of MHPSS programmes has gradually developed over the last 25 years [6,21]. One recent systematic review reported that, out of 24 reviewed programmes, 60% conducted assessment adaptations and 40% conducted programme adaptations; the locally-adapted programmes showed better effectiveness than those without adaptation [6].

By applying cultural adaptation, the biomedical approach seems to have reduced the gap between MHPSS programmes and local needs. However, the localising of Western-origin programmes is still top-down and expert-driven, and neglects local healing processes and resilience; a situation driven by the underlying unequal international power relationship. To resolve these issues, the psychosocial approach has been developed [22,23,24,25]. This approach respects local resilience and puts the local community at the centre of the whole cycle of MHPSS. For example, Participatory Action Research (PAR) in Sierra Leone, Liberia and Northern Uganda is a pioneering project based on this approach [26,27]. Researchers included participants in the project cycle from the planning stage and let them define the problems they faced, plan and implement activities to address their problems, and evaluate their achievements themselves. Similarly, sociotherapy in Rwanda, which is widely acknowledged as effective by local communities, also facilitates the community’s mutual decision-making regarding which problems they wish to cope with and how [28,29]. Importantly, both projects entrust the community with deciding which problems to tackle and how to resolve them, instead of imposing internationally-defined outcomes and intervention programmes.

As represented by the psychosocial approach, the most recent MHPSS in war-affected populations explores resilience-oriented and community-driven (bottom-up), rather than deficit-oriented (trauma-focused) and expert-driven (top-down) approaches [24,25]. However, knowledge of community resilience––e.g., local healing processes, assets, and resources––and how collaboration can be achieved is still scarce. Participatory Action Research and sociotherapy, as discussed earlier, may be examples of successful MHPSS collaboration with local communities but information from these studies was limited to experiences in the communities which received the support. Most importantly, prior studies have not paid sufficient attention to the reality that MHPSS recipients often live in larger communities where many others do not receive the same support and that they are interacting not only with the support programme but also with others from local communities. To improve collaboration with local communities, we also need to know about interactions between those who receive MHPSS and those who do not, and the long-term consequences of MHPSS for their relationship.

### Context of Musanze, Rwanda

Rwanda is one of the representative countries that have fed debates surrounding MHPSS provision [2,11,13,16]. Although it has received various forms of international aid support, including MHPSS, since the 1994 genocide, some regions of the country have not had sufficient support for recovery. This research sheds light on the resilience of one such region, the Musanze district in the north, and how MHPSS recipients interact with other local citizens.

Rwanda experienced a series of civil wars and genocide between 1990 and the year 2000 [30,31,32]. Of these, the most commonly known to the international community is the genocide against the Tutsi in 1994, which occurred at the end of the preceding war (1990–1994). During the genocide, Tutsis and moderate Hutus were massacred by the former Hutu-led government and Hutu militias. The Rwandan Patriotic Front (RPF), a Tutsi-led rebel force, ended the genocide and took over the country in 1994. However, after that, between 1997 and 2000, the region of Musanze went through another event, the insurgency in the northwest, locally called the *abacengezi* war (pronounced *abacyengezi*, meaning ‘infiltrators’). The inhabitants of the region, mostly Hutus, were massacred during the *abacengezi* war [32,33,34]. Although no official data on victims of this tragedy are available, the district survey traced its impact eight years after the end of the insurgency; 21% of children in Musanze were orphans [35] which was 5% higher than the national average [36].

International aid organisations have provided a number of MHPSS programmes to respond to the 1994 genocide and support genocide survivors. These programmes include bio-psychological and psychotherapeutic programmes [12,37,38,39], resilience-oriented psychosocial programmes [29,40], and other creative activities for mental healing and reconciliation [38]. Meanwhile, victims of the insurgency in the northwest have been marginalised. International as well as government support to Musanze is extremely limited since those in the population are mostly victims of the insurgency, not survivors of the genocide. Despite the limited support, Musanze has shown remarkable social-economic growth over the last two decades; the percentage of the population that was poor was nationally ranked second lowest after the capital Kigali in 2010 [36]. In short, the majority of Musanze citizens have recovered from the insurgency in the northwest with very little aid. However, there are a few genocide survivors in Musanze for whom there is MHPSS. This research then explores how the two different groups interact with each other and how they perceive and experience the mental health impact of the atrocities and the healing process.

## 2. Objectives

Given the discussions above, this research focuses on Musanze, Rwanda and aims to explore:

(1) How MHPSS recipients interact with others from local communities who do not have any support; and
(2) The gap between MHPSS programmes and local communities which do not have any support in terms of perceived mental health impact of atrocities and healing processes; and how the gap could be filled.

By exploring the above, this research attempts to contribute to the improvement of MHPSS programmes and the development of collaboration between MHPSS and local resilience.

## 3. Methods

The qualitative research, including in-depth interviews, focus-group discussions (FGDs), and participant observations, was conducted in the Musanze district between August 2015 and May 2016 and built on my prior life and work experience in the field over two years. I conducted the research with the assistance of local residents from Musanze. Research techniques from grounded theory [41,42] informed the whole research cycle of sampling, data generation, analysis, and writing.

### 3.1. Research Participants and Sampling Strategy

A total of 43 local residents participated as individual interviewees, having given informed consent. Since three did not complete interviews for fear of political consequences, data from 40 participants (24 women and 16 men, aged 22 to 84 years) were analysed (Table 1). Some participants were repeatedly interviewed or were asked to participate in FGDs to collect further information based on theoretical sampling. Information gained from informal interviews, which were part of the participant observation, also contributed to the analysis. The observation focused on everyday life and social activities (e.g., community meetings and community work). Data for analysis included fieldnotes, interviews, and FGD transcriptions. All participants were given two kilograms of rice (worth approximately GBP 1.20, August 2015), which provided two days’ food for a family, as an honorarium for participation.

To collect common narratives of suffering and healing experience among local residents of Musanze, participants were approached in villages through networks that my assistants and I had already established. In the fragile post-war context, trust built on existing networks was vital to gain access to the research site, participants, and their stories. Sampling was conducted in two stages, initial and then theoretical sampling until the analysis reached theoretical saturation according to grounded-theory approach [42]. Initial sampling began in the local area and gradually involved neighbouring areas to include a maximum variation sample of participant characteristics, including age, gender, former ethnicity (as the government’s ‘one Rwanda for all Rwandans’ policy disallows divisions on ethnic lines, all references to ethnicity in this paper relate to pre-genocide identities), occupation, socio-economic status, and home village, in order to facilitate analysis of experience of suffering and healing. After the initial analysis, the research moved to theoretical sampling which sought relevant data to examine the coding schemes, analytical questions, and a provisional hypothesis emerged from the initial analysis. The coding schemes were developed and refined through the cyclical process of theoretical sampling and analyses through constant comparison and memo-writing. The sampling process was terminated when information provided by new participants began to exceed the research scope (community resilience), not just repeat the obtained data, which shows that the research had reached ‘saturation’ [42].

### 3.2. Data Generation and Analysis

Almost all the interviews and FGDs in Kinyarwanda (four interviews were in English). Although the author speaks Kinyarwanda, a local assistant acted as interpreter during the interviews since interviewees suspected that the author might be a government inspector or be working for Hutu extremists when she conducted interviews in Kinyarwanda by herself. It was necessary to gain access to potential participants through the author’s networks and those of local assistants based on trust. Interview topic guides were produced through close discussions with local assistants, tested and refined for local adaptation before use. They were designed to be loosely structured and conversational, using three questions to facilitate story telling about suffering and healing (Table 2). Interviewees were told to feel free to say whatever they wanted for as long as they needed. The number of key questions, probes, and interpretations during the interview were minimized to prevent interruption of stories. The author made notes on non-verbal narratives and contextual information without disrupting interviewees’ storytelling.

All interviews were transcribed and double checked by the author and assistants according to agreed guidelines. One assistant translated the Kinyarwanda transcriptions into English, after which the author checked and refined them in conjunction with another assistant and produced the final translation. While producing translations, assistants provided rich cultural and contextual accounts which the author transcribed and analysed as data. Data was analysed constantly through the lifecycle of the ethnography, developing coding schemes manually and refining them through constant comparison and memo-writing. To ensure representation of participants’ subjectivity, ‘member check’ [42] was conducted with local assistants and participants.

### 3.3. Ethical Considerations

The study was approved by the Rwanda National Ethics Committee (No. 339), the Ministry of Education (No. 2944) and the Ethics Committee of the London School of Hygiene and Tropical Medicine (LSHTM Ethics Ref: 9182). All quotations are anonymised and pseudonyms are used in this paper.

## 4. Findings

The findings first present a case study about the suffering of a Tutsi genocide survivor who is supported by MHPSS, and then discuss why she suffered despite the support, showing the gap between local communities and MHPSS in terms of conceptualisations of suffering and healing processes (see also Otake [43]). Finally, in the light of local practices, I will explore how the gap can be filled.

### 4.1. Suffering of Those Who Are Supported by Mental Health and Psychosocial Support (MHPSS)

Since 1994, the Tutsi-led government and government-affiliated national and international organisations have focused on supporting genocide survivors and returnees, who are mostly Tutsis. The representative organisations include The Genocide Survivors Support and Assistance Fund (FARG), *Association des Etudiants et Éleves Rescapés du Genocide* (AERG), and AVEGA-Agahozo, which are locally managed but backed by international aid organisations and the government to support genocide survivors. Their support thoroughly covers survivors’ livelihoods, providing psychological counselling, medical services, financial support for education, income-generating activities, housing and legal services [38,44,45,46]. Likewise, a district officer in Musanze told me that the government provides genocide survivors with psychological counselling, financial support for school fees, income-generating activities, and FRW (Rwandan Franc) 10,000 (approximately GBP 10, August 2015) monthly. For returnees, to my knowledge, the government, as a minimum, builds houses and provides financial support. Other international non-governmental organisations additionally provide genocide survivors with trauma healing and reconciliation programmes.

This assistance has supported victims to survive and recover from the genocide. However, some genocide survivors in Musanze, particularly those who arrived after the war period and did not share the massacre experience with local people, suffered serious social isolation and lack of understanding from their neighbours. The following account of Murakatete represents such suffering.

#### Social Isolation of Murakatete, a Tutsi Genocide Survivor

Murekatete is a Tutsi woman in her late 20s. She is originally from Kigali and came to Musanze to study at university four years ago. She lost all her family and cohabiting relatives except her mother during the 1994 genocide. Since then, she has received various forms of support from both governmental and internationally-backed local organisations such as FARG and AERG. Murekatete told about the socio-economic support from FARG:

FARG paid my school fees until graduation, even though I haven’t got the opportunity to further my studies in higher education. [… ] FARG has been on our side and we did not lack anything including school uniform, notebooks, ticket fare for students living far away. The money was sent to us. Also, in the holidays, food was reserved for children without parents. There was food to eat until schools reopened. Orphan students were equally given a home where they could spend holidays. (Interview 16 March 2016)

Murekatete regarded FARG and AERG as the only sources of support in her life since the genocide. She has a strong connection to those organisations and does not feel close to any other people in the local community. She also recounted her experience of participating in the MHPSS programme in AERG, which emphasises ‘talking’ about traumatic experiences:

[In the AERG meeting] you feel that your mind is released because of talking about such issues [suffering resulting from the genocide]. […] Indeed, when we talk with someone with whom we share the same problems, we feel secured in our minds. (Interview 16 March 2016)

For her, members of AERG share the same suffering as they are all genocide survivors. They can talk freely about their genocide experience and the resultant suffering in the meeting, which allows her to heal.

However, notably, despite the socio-economic, mental health, and psychosocial support, Murakatete experienced serious suffering because of isolation from the local community:

I have been living here for four years, but I have not been able to be sociable with other people. In fact, I do not do so. I ask myself what I can talk about with them. […] Our lives are not similar so I haven’t been able to feel confident with others. I can say that here in the quarter I haven’t been able to make friends because I think that no one can help me solve my problems. Therefore, for me there is nothing we can talk about. (Interview 16 March 2016)

Murakatete believed it was useless to talk about her own suffering with her neighbours. She was in fact unable to share her genocide experience through ‘talking’ with local residents; even if she did talk, they did not really understand her. Thus, she became withdrawn and isolated from the local community. In other words, ‘talking’ about the genocide was effective in healing her within the MHPSS group but isolated her within the local community.

She has continued to receive support from the same aid organisations as before her move. Nevertheless, she suffered from social isolation in the village she moved to where the majority of residents are Hutu victims of massacres during the *abacengezi* war. Other genocide survivors who moved to the village after the war period also expressed feelings of isolation and lack of understanding by their neighbours, particularly in community meetings during the genocide memorial week. By contrast, Tutsis who were originally from the village and shared experience of massacres during the *abacengezi* war were generally perceived by other residents as part of the community. Amongst those who had recently moved to Musanze, Hutus tended to be integrated through social groups such as faith-based groups and mutual-saving groups. The isolation of Murakatete and other genocide survivors was thus likely to have arisen from a combination of ethnicity and genocide experience unshared with local residents.

### 4.2. Local Conceptualisation of Suffering

Murakatete’s social isolation in the local community was not, however, simply due to her ethnicity and genocide experience being different from majority of local residents. To explore the underlying reasons for her isolation, in Section 4.2 and Section 4.3, I will explain the conceptualisation of the mental health impact of massacres (which I alternatively call ‘suffering’) and the healing process in Musanze communities. 

#### 4.2.1. Ibikomere (Wounded Feelings)

To date, researchers of post-genocide mental health in Rwanda have acknowledged the word ‘*ihahamuka*’ as the local translation of Western bio-psychological ‘trauma’. It refers to breathlessness, ‘breathless with frequent fear’ [47] and originated from African root words that indicate the absence of inhalation or a state of not breathing [48]. The word was improvised by genocide survivors to express the bio-psychological impact of the genocide they had experienced in line with the Western ‘trauma’ concept; however, it did not exist in the local community until international aid organisations began to deploy MHPSS programmes following the genocide [16,48,49,50]. Over the last 20 years, MHPSS have taught local Rwandans ‘*ihahamuka*’ through psycho-education, campaigns, trauma healing and reconciliation programmes; the main target populations of these programmes were genocide survivors.

However, most of my research participants have been excluded from such programmes because they are not genocide survivors but victims of massacres during the *abacengezi* war. Therefore, with the exception of the few who had been trained on post-genocide trauma at school or by international aid organisations, participants were unfamiliar with the word ‘*ihahamuka*’. Even if they had heard it, they thought it was for genocide survivors and not for themselves. To express their own suffering, instead, they used the word ‘*ibikomere*’ (plural)/’*igikomere*’ (singular), meaning ‘wounded feelings’.

The reported *ibikomere* included various feelings; for example, being sad (*kubabara*), deep sorrow (*intimba*), depression (*agahinda*), hopelessness/despair (*kwiheba*), being anxious/worried (*guhangayika*), fear (*ubwoba*), and mistrust (*kwishishya*). However, the most common *ibikomere* feelings of social isolation and grief––i.e., isolation, loneliness, and helplessness, due to the loss of family and relatives. The story of *ibikomere* very frequently began with an episode of family loss and then described a feeling of being left alone. For example, one man described his refugee experience in the Democratic Republic of the Congo (DRC) when he was 15 years old. He lost his uncle who took care of him and many others who shared his refugee life while fleeing through a forest. Looking back at that time, he recounted his feelings of loneliness and helplessness;

*Ibikomere* that I remember, for me… I can say, the time in Congo [DRC] […]. I suffered… because I was alone and also someone who… can help me, like my uncle, also died in that period. So I stayed without anyone who can help me. (Male, 30s, interview 10 May 2016)

Another woman told about her experience of surviving massacres during the *abacengezi* war, her feelings of isolation and deep grief:

*Igikomere* that I will never forget is… Can you imagine that you had lived with many neighbours and you see all of them were killed and stay alone in that area? I can never forget this situation in my life. I never forget that I had all of my parents [including elder relatives and neighbours], but few of them survived. Many siblings and friends died and I stay… I stay with few of them. I have only few of them survived. (Female, 40s, interview 16 December 2015)

As shown in those narratives, local communities experience and understand the mental health impact of massacres in terms of social isolation and grief resulting from the loss of loved ones.

#### 4.2.2. The Development of Ibikomere Due to Social Isolation

Participants explained that *ibikomere* develops toward a more severe mental status; ‘*ihungabana*’ (mental disturbances), then ‘*ihahamuka*’ (trauma), and finally ‘*kurwara mu mutwe*’ (having an illness of the head) (Table 3). One concept develops into another which is slightly more serious than the last, while the *ibikomere* feelings are sustained as emotional difficulties throughout this development.

The development of these symptoms was characterised by the degree to which participants experienced social isolation and how far their thoughts and memories were oriented towards the past. That is, the more participants experience isolation from society, the more they are haunted by memories and thoughts on the past; and their symptoms become more severe. This development process was also reported as a vicious circle of social isolation, ‘remembering’ and ‘thinking too much’ about the past. Generally, participants described *ibikomere* as arising when they remember and think too much about their deceased family members; for example, “if someone remembers it, *ibikomere* also comes” (male, 50s, interview 9 April 2016) and “I always think about them [lost family members] and it makes my *ibikomere*” (female, 70s, interview 31 March 2016). Such remembering and too-much-thinking are initiated and also reinforced when they are isolated from society. Explaining how *ibikomere* can develop *ihungabana* and *ihahamuka*, a young woman said;

If you stay alone here [at home], you cannot help but remember it. […] When I am with many people or when I am talking to some people, I don’t remember a lot. But when I am alone, I think about my life [and *ibikomere* becomes more severe]. (Female, 20s, interview 29 November 2015).

Similarly, *kurwara mu mutwe* can also develop through social isolation and too much thinking. Another woman explained her neighbour’s *kurwara mu mutwe* symptoms in a focus-group discussion (FGD): “being withdrawn and living alone, they are all combined. Maybe this [combination] is the cause of her *kwiruka* [running; a symptom of *kurwara mu mutwe*]” (Female, 40s, FGD 21 December 2015). Another man in the same focus group added: “When you are withdrawn, the brain starts thinking too much and cycling too much. After that the brain becomes to be like broken” (Male, 30s, FGD 21 December 2015).

In short, local conceptualisations of the mental health impact of massacres during the *abacengezi* war emphasise social and emotional aspects of wounds, where isolation is key to initiating and reinforcing ‘remembering’ and ‘thinking too much’ about the past, resulting in the development of mental health problems. This can contrast with the Western trauma concept, locally translated as *ihahamuka,* which focuses on the individual’s bio-psychological symptoms of breathlessness and fear. Given the gap between perspectives, genocide survivors who understand their suffering as bio-psychological *ihahamuka* may not be able to share their experience with local residents who understand their suffering as social *ibikomere*.

### 4.3. Local Healing Practices

Whereas social isolation plays a key role in developing the locally-perceived mental health impact of massacres during the *abacengezi* war, social reconnection opens a window to the healing process in local communities. Although most participants have never been involved in MHPSS, they reported healing experiences within different social groups in local communities. The most common social groups that were reported to be therapeutic were church-based groups, mutual-saving groups, and kinship and neighbourhood groups. Church-based and mutual-saving groups are based on neighbourhood relationships; participants generally took part in multiple groups, rather than belonging to only one. In this section, I will explain the ways in which participants heal the social wounds of massacres through participating in these groups (see also Table 4).

#### 4.3.1. Talking for Reconnection

One important healing practice in local communities is ‘talking’; however, this is different from that in MHPSS. As described in Murakatete’s case, the main component of many MHPSS programmes is talking about traumatic memories and trauma-related problems in life. This is because MHPSS programmes, particularly those which apply ‘evidence-based’ practices [6,7,8], have the theoretical foundations of psychiatric ‘talking cures’ and/or psychotherapeutic techniques, such as cognitive behavioural therapy (CBT) [51,52]. They aim to transform the traumatic memory by talking, so that it can be integrated into a personal life history. In this research, too, participants frequently mentioned ‘talking’ as an effective and significant healing practice within their communities. However, unlike MHPSS, the purpose of their ‘talking’ was ‘social reconnection’, rather than cognitive transformation of traumatic memories.

#### *Gusura na Kuganira* (Visiting and Talking)

Talking for reconnection was mainly referred to as ‘*gusura na kuganira*’, meaning ‘visiting and talking’ to others. For participants, it was a series of activities which build and maintain social connection with neighbours and other community members in everyday life. One notable activity of *gusura* (visiting) is provided by church-based groups; they visit those who have withdrawn after family loss in order to help their social reconnection. For instance, a woman from the Adventist church, who lost all her family and kin members during the war period, said: “I was always withdrawn and having problems, then they [church members] came and taught me [the word of God] and comforted me. [… Then] the patience came [to me] little by little” (Female, 30s, interview 7 November 2015). In her description, visiting naturally leads to talking activities, such as comforting; this combination allows withdrawn people to be reconnected to society.

It is noteworthy that for participants, *kuganira* (talking) rarely referred to talking about trauma. When they reported the healing impact of *kuganira*, they mostly referred to talking with each other without necessarily talking about their war experience. For example, in church-based groups, *kuganira* generally draws on words and episodes from the Bible, which allows members to comfort each other. According to a woman from a Catholic church group;

When we finish praying, then we talk together about some episodes or the word of God [from the Bible]. We read and learn the word of God. It helps us to meet different people or other Christians. Also, we talk over the words of God to comfort each other. (Female, 30s, interview 8 April 2016)

Meanwhile, *kuganira* in mutual-saving groups refers to discussions on how to resolve problems in everyday life and in small businesses. One group member explained how it heals members’ wounds:

In a mutual-saving group, when you are with others, you discuss with others and reach a mutual conclusion [to resolve an everyday-life issue]. Someone speaks their ideas and others bring discussions; this helps people to forget [the past. …] and you feel better in the heart. (Female, 30s, interview 8 April 2016)

As noted above, members of these groups generally talk to each other about the Bible or resolving everyday-life matters. What cuts across these different styles of *kuganira* is the growing awareness that “I’m not the only one” who suffers. As recounted by a member of mutual-saving group who lost both parents during the war; “When you talk to others [in a meeting, …] you understand that you are not alone and you are not suffering alone” (Female, 40s, interview 20 April 2016). These narratives are revealing: even though participants do not necessarily talk about their traumatic experience directly, reconnection for healing can take place through alternative narratives, such as Bible discussion or everyday issues.

#### 4.3.2. Living Together

Social reconnection as described above enables people to experience more profound healing and reconciliation through sharing and helping each other in everyday life. This small section explains major healing practices that recovers collective lives: *gusangira* (sharing), *gufashanya* (helping each other), and *kwiyunga* (reconciliation).

#### *Gusangira* (Sharing) and *Umusabane* (Social Party) 

One important common theme of local healing practice is *gusangira*, which refers to ‘sharing’ things, such as food and drink, everyday life, ceremonies, life-stories and memories with family members, neighbours and friends. The concept very frequently appeared in interviews and community observation. As recounted by an elderly man; “for example, you think about sharing something (*gusangira*) with a person who used to be close to you, and you realise that you are no longer with him or her. […] You see that it is also *ibikomere*…” (Male, 50s, interview 9 April 2016). *Gusangira* was described as an important element of the life they lost because of the war and thus needed to be retrieved.

Although *gusangira* is observed in various everyday settings, the most symbolic one is practiced in *umusabane*, a ‘social party’, which is held at the end of mutual-saving group meetings or as part of life-event ceremonies. A member from a mutual-saving group talked about *umusabane* in his group:

[In a meeting] we converse and give money. We just make a regular contribution every week, and then we discuss issues. [If] some members want [to take] loans, leaders give it. [Also] leaders distribute money to members whose turn it is to take money. […] In particular, after finishing [the financial transactions], we do *umusabane*. We drink beer together. (Male, 20s, interview 17 November 2015)

Generally, traditional mutual-saving groups have similar meeting schedules and have *umusabane* after all the financial transactions. While drinking local banana beer in *umusabane*, members share problems and discuss how to resolve them together. In my observation, they coped with any livelihood issues, for example, improving their business, paying tax, catching thieves, fixing a house, children’s education, and planning life-event ceremonies. Sometimes group members took action to help individual members resolve their problems. In short, *umusabane* is not only an opportunity for sharing drinks but also sharing everyday problems and helping each other cope with them. For participants, this mutual help heals their wounds. For one mutual-saving group member; “The group plays a role in mental recovery because when you meet a lot of people [in a meeting], you talk to them and they give you advice on tomorrow’s life” (Male, 20s, interview 17 November 2015).

#### *Gufashanya* (Helping Each Other) and *Umuganda* (Community Work)

‘Helping each other’, as in mutual-saving groups, is called *gufashanya* in Kinyarwanda; it was also observed in church-based groups and neighbourhood relationships. In fact, *gufashanya* was the most frequently occurring theme throughout the research as it is the most important social and moral norm for local Rwandans. In a religious and spiritual context, the word *gukundana* (loving each other) is also frequently used, referring to mutual help. Some participants saw that Musanze citizens helped each other more after the war. For the leader of a mutual-saving group: “After the problems we experienced here in Rwanda... many people began to think more [about consequences of the war] and there were situations which required us to love and help each other” (Male, 40s, interview 22 November 2015).

Like *gusangira*, *gufashanya* is also practiced in various scenes of everyday life; common practices of *gufashanya* include helping with farm work, housework and child care, sharing food, water, firewood and other livelihood materials, and providing financial assistance within the community. However, the most symbolic activity of helping each other is *umuganda*, which refers to ‘community work’, frequently farm work, to help vulnerable members. The history of *umuganda* predates the arrival of Christianity in Rwanda. According to one elderly woman; “Many years ago, before churches started preaching everywhere, […] neighbours were to help vulnerable people. […] It means that it is the culture we have had in Rwanda [since before Christianity]” (Female, 50s, FGD 21 December 2015).

In particular, when someone is vulnerable––e.g., they are sick, pregnant, old or poor, it is perceived as morally important to help that person and his/her family, so the community provides *umuganda*. During the research period, I observed a church-based neighbourhood group conduct *umuganda* for a sick elderly woman. One of the group leaders explained that it is a communal effort for survival and prevention of social isolation. He said: “*Umuganda* is very important because, for example, if you don’t give someone *umuganda*, she cannot survive. But if you give it to her, she becomes happy because she can be with other people. She doesn’t feel sorrow” (male, 60s, interview 19 December 2015). Another group member also recounted why they help her:

We [neighbours] are like one family. [… This is why] we cultivate for her, give her water, sometimes give her firewood, and whatever we find. [… If we help her] we will be able to see that she will survive and move her days. (female, 40s, interview 21 November 2015).

The group also weeded the elderly woman’s farm; then commenting on their *umuganda*, she described her healing experience:

Although I am ill, they helped me very much. I feel relieved. They do what I cannot do, then do you think that I’m not relieved? … I’m relieved. [Since] they worked for me, I’m relieved. […] Of course, it is recovery of *ibikomere*. (Female, 70s, interview 21 November 2015)

Nowadays this traditional means of community work is taken by the government and implemented as the government-led nationwide ‘*umuganda*’. This has top-down schedules, obligatory participation and aims to rebuild the country. By contrast, the community’s *umugada* is based on grassroots decision making and voluntary participation. It is driven by the local norm of *gufashanya* and aims to help vulnerable members. It is thus experienced as more therapeutic than the government approach.

#### *Kwiyunga* (Reconciliation) and *Umuvugizi* (Mediator) 

For Musanze citizens, *kwiyunga*, or ‘reconciliation’, is the most crucial part of the healing process. Life in a village requires residents, whether victims or perpetrators of an incident, to continue to live together and help each other for survival. Even though it is challenging, victims wish to reconcile with perpetrators because for them, perpetrators are often part of their life-history and even collective identity, such as family and kin members or old friends with whom they have shared important memories from before the war. For them, a village is like a ‘large family’, where members are to live in harmony.

The traditional concept of *kwiyunga* is generally characterised by the following process: first a perpetrator asks a victim for forgiveness, then the victim accepts it and forgives. If the perpetrator does not come to ask for forgiveness, the victim can visit the perpetrator to ask for an apology. When both perpetrator and victim are afraid of meeting in person, they can involve a ‘mediator’, called an ‘*umuvugizi*’, such as a religious or community leader or a mutual friend. This style of reconciliation is possible with genocide survivors whose perpetrators are clearly identified; some local and international non-government organisations are supporting the process. However, such support is not available for Musanze citizens because they do not know who the perpetrators were or even if they know, they cannot name them due to the political constraints surrounding the *abacengezi* war. They can neither ask perpetrators to apologise nor expect them to come to ask for forgiveness. Nevertheless, many reported their *kwiyunga* experience with those anonymous perpetrators; how do they process it?

Their *kwiyunga* in fact goes on in everyday life, through living with others in village. Moreover, *kwiyunga* for them did not mean reconciliation with only perpetrators but also all human beings. Under circumstances whereby people cannot identify killers who may live with them as neighbours or even as family members, they can feed a wider sense of mistrust against all humans, thus generating trust and reconciliation with all human beings becomes a significant theme. For instance, when community members have a conflict for any reasons due to mistrust, mediating it through an *umuvugizi* mediator is important. A leader of a church-based group recounted her experience of mediating conflicts related to land occupation among members:

There was a person who let his chickens come into the neighbour’s farm so the chickens were eating the neighbour’s plants. I told the chicken owner that he should stop allowing his chickens to come into his neighbour’s farm and that he should keep them around his own place. He understood and followed my advice. I went to do follow-up of the case and saw that the neighbour’s beans are growing well. The chicken owner never did it again. We have done [mediating] activities like that. (Female, 40s, interview 6 November 2015)

Even when they have no conflict, the *kwiyunga* process continues by simple participation in social activities, particularly when people who have different backgrounds, including ethnicity and religion, take part. For example, one woman explained that mutual-saving activities connect different people and promote reconciliation:

Due to the history we went through, it is possible that some [of the group members] may have plotted against my family. Someone may have betrayed my family and all of them got killed, which left me completely alone. So you understand that it is difficult to sit together again and talk to each other. It is a difficult thing. But the mutual-saving group tries to teach and bring us together, so that no one can continue to think of another one as one’s enemy. (Female, 20s, interview 26 March 2016)

Outside social groups, their *kwiyunga* continues through living with others in their village. An elderly man reported:

If I live with my neighbour and he has any problem, like having a sick family member, it is good to help him even if that person is like my enemy. When other people go there [to help him], I can’t stay at home. Those are the things that help my heart to feel well. (Male, 60s, interview 9 April 2016)

As shown in these narratives, local reconciliation processes take place in social systems that are already in place within communities, while sharing everyday-life activities. Such reconciliation allows people in villages to reconcile not only with perpetrators but also with all human beings.

#### 4.3.3. Praying Together

Alongside community healing in everyday life settings, the healing process also takes place through religious and spiritual practices. These aspects of healing were commonly described using the word ‘*gusenga*’, meaning ‘praying’. The word has multiple meanings and can be used with other healing concepts. For example, when *gusenga* is used with *gusura* (visiting), it means an outreach activity by church-based groups; with *kuganira* (talking), it suggests conversations citing episodes from the Bible in a church-based group meeting; with *gufasha* (helping), it refers to charity activities to help vulnerable people in villages; and with *kwiyunga* (reconciliation), it signifies individual prayer to reconcile with perpetrators in one’s heart with the help of God. Except when used with *kwiyunga*, *gusenga* means communal, rather than individual, prayer.

*Gusenga* was mainly reported by participants who belong to church-based groups; however, those who were outside these groups also perceived religious and spiritual healing as very important since they understand their survival in relation to God. Participants often used phrases such as ‘*impano y’imana*’ (a gift of God) and ‘*kubera imana*’ (thanks to God) to express their spiritual appreciation when they narrated their survival. Generally, Musanze citizens believe in God’s protection in all aspects of life; therefore, ’God’ (*Imana*) was one of the words that I heard most frequently in everyday settings during my research.

Overall, participants who have never been involved in MHPSS were coping with the mental health impact of massacres by participating in social groups and through everyday life practices in local communities. Communities and social groups healed members by promoting reconnection, providing social and spiritual support and mediating reconciliation. Compared with MHPSS programmes, local healing processes placed much more emphasis on social aspects of recovery and on healing through ‘practicing’ and ‘living’ everyday life with others, than ‘talking’ about traumatic experience. This may be one notable reason why genocide survivors in bio-psychological MHPSS cannot share the narrative of healing with local residents; Murakantete, who I presented earlier, said “there is nothing we can talk about”. Moreover, genocide survivors do not actually need the community’s support to survive; instead, the socio-economic assistance that accompanies the MHPSS supports their livelihoods thoroughly. Consequently, they are outside local support networks, relying more on MHPSS networks and socio-economic aid packages. In this way, MHPSS recipients can become isolated within local communities.

### 4.4. Integration of Those Who Are Supported and Unsupported

So far, I have demonstrated local conceptualisations of suffering from war and healing practices and discussed the gap between them and bio-psychological MHPSS. How then can this gap be filled? How can the isolated MHPSS recipients be integrated into local communities? In this section, I will explore answers to these questions, drawing on narratives from two Hutu massacre victims from Musanze, Dieudonné, and Muhoza.

#### 4.4.1. Sharing Suffering Experiences

Dieudonné is a young Hutu man in his late 20s who lost his parents during the *abacengezi* war. He was originally from Musanze, then studied in the Huye district, in the south province, where the local population experienced the genocide against the Tutsi. His circumstances were apparently similar to Murakatete in terms of living in an area where local citizens did not understand his massacre experience and suffering. However, unlike Murakatete, he experienced reconciliation and friendship, rather than isolation. One day he had a chance to talk about himself to one of his roommates who was a Tutsi genocide survivor from the local area and this resulted in developing friendship with him:

He lost his both parents during the genocide against the Tutsi in 1994. He told me that all his family and relatives were killed and […] he’s been helped by neighbours [with his survival]. […Because he shared something difficult for him with me, in return] I tried to share my past experiences with him. I told him; “even me I am an orphan. My father was killed during 1997 because of this [*abacengezi* war].” [… Then] he told me: “Dieudonné, I like you. I like how you behave. […] I and you shall be strong. We shall have a good life in our future.” […] I also advised him how he can behave [so that] he can succeed very well. […] We were the same cases, you see. His parents died during the genocide but my parent died during *abacengezi*. But we helped [each other]. Even now we help each other. (Interview 12 May 2016)

#### 4.4.2. Sharing Life

Dieudonné’s experience suggests that sharing difficult experiences through ‘talking’ to each other can open a window to reconciliation and integration between a Hutu massacre victim who does not receive MHPSS and a Tutsi genocide survivor to whom this support is given. Muhoza also reported a similar experience in Musanze; she is a young Hutu woman in her 20s who lost both parents and relatives during the *abacengezi* war. She met a Tutsi genocide survivor, Odette, at her workplace in Musanze and developed friendship with her. While acknowledging the importance of ‘talking’ to each other, she also recounted the healing impact of ‘sharing life’:

First we should start with talking to each other and after that I can tell her about myself. [Then] we heal each other by sharing our life and caring for each other. […] She was a genocide orphan. She lost her parents during the genocide. She was living alone. We were the same. The difference between us was that she was an orphan of the genocide [and I am an orphan of the war]. (Interview, 29 November 2015)

Muhoza said that after they became good friends, Odette got engaged to a man living in Kigali. Although she moved back to Kigali, she invited Muhoza to her wedding which would take place a week after the interview. Muhoza said: “I will go there because I want to see and greet her”.

Dieudonné and Muhoza’s narratives suggest that social integration between a MHPSS recipient Tutsi and a non-recipient Hutu can happen through ‘talking for mutual understanding’, then ‘sharing’ and ‘helping’ in each other’s lives. ‘Talking’ here is slightly different from both talking about trauma (MHPSS practice) and talking for reconnection (community practice). When reconciliation happens, they talk and also listen sympathetically to each other’s story, leading to mutual understanding of different experiences. Through such conversations, the isolated sufferers can become involved in the community healing practice, ’living’ together.

## 5. Discussion

This qualitative study revealed the social isolation of a MHPSS recipient who lives in a local community in Rwanda, where the majority of residents do not receive such support. Exploring the reasons for the isolation, the research has highlighted three significant gaps between MHPSS programmes and local communities, regarding perceived mental health impact of atrocities and the healing process. First, whereas many MHPSS programmes apply bio-psychological frameworks to understand post-genocide mental health and healing processes, local communities emphasise social aspects. Second, MHPSS programmes encourage ‘talking’ about traumatic experiences and trauma-related problems. By contrast, in local healing processes, social support plays a major role and healing takes place through the ‘practice’ of everyday life and ‘living’ together. Third, MHPSS programmes are designed to facilitate healing within intervention groups in which people who have the same background are gathered; however, healing in local communities takes place in the course of everyday life, through interaction and mutual support among different people. Despite these gaps, the research also found that ‘talking for mutual understanding’, then ‘sharing’ and ‘helping’ each other in an everyday setting can fill the gaps, resolve the social isolation of MHPSS recipients, and open a window to integration into the local communities. In the remainder of this section, I discuss these gaps and how MHPSS programmes could be improved and work in collaboration with local resilience.

### 5.1. Mental Health Impact of Atrocities: Bio-Psychological Trauma Versus Social Wounds

In this research, participants from local communities which do not have MHPSS commonly conceptualised the mental health impact of massacres during the *abacengezi* war as *ibikomere* (wounded feelings), which are represented by feelings of social isolation and grief resulting from the loss of loved ones. The concept of *ihahamuka*, the local translation of Western-origin bio-psychological trauma, was only reported by those who had received MHPSS interventions or had been trained in trauma as health intervention facilitators. This finding reiterated previous studies from other conflict-affected settings in terms of reporting social isolation and grief as the most common local experience of war-related mental health problems [25,53,54]. Also, the finding that the concept of *ihahamuka* is not familiar to local communities which have never received MHPSS echoed Bolton’s [16] study in Rwanda. Kleinman [55] raises the possibility that global health interventions among conflict-affected populations can transform local conceptualisation as well as experience of suffering. However, this research showed that despite nearly two decades of nationwide interventions by international aid organisations, local communities preserve their ways of understanding suffering whilst the influence of the intervention was also observed among a few community members. MHPSS programmes that teach ‘trauma’ tend to focus on supporting genocide survivors, who are mostly Tutsis. Therefore, other community members, who are mostly Hutus, were unfamiliar with the ‘trauma’ concept or thought of it as only for genocide survivors; instead, they preserved their local concept. In this context, the ‘trauma’ concept is closely connected to Tutsi genocide survivorship and possibly play a role in increasing the isolation of genocide survivors and even ethnic division within local community.

### 5.2. The Healing Processes: Talking about Trauma Versus Living Together

As the locally-conceptualised *ibikomere* emphasises social aspects of mental health issues, the local healing process is also characterised by social reconnection and recovering social fabric in everyday life. The community healing practices are most commonly represented by ‘sharing’ (*gusangira*), ‘helping each other’ (*gufashanya*), and mediation of ‘reconciliation’ (*kwiyunga*). Seeing this as social support, studies of social support and health [56,57] provide theoretical justification that they lead to a recovery of mental health and wellbeing. However, I also interpret it as a practice of ‘living together’ in the light of prior ethnographic works which propose a notion of ‘living’ as a symbol of healing practices in war-affected communities [50,58,59]. In their observation, engaging in everyday-life practices, such as livelihood activities, work, ceremonies and religious activities, constitutes a healing process; through ‘living’, survivors reconstruct their life itself and thus it becomes healing. My findings extended this notion of ‘living’ by shedding light on the collective lives that are being lived and recovered.

This paper contrasts the community’s practice of ‘living together’ with the ‘talking’ practice which is the main component of many MHPSS programmes. MHPSS often facilitates talking about traumatic experience and trauma-related problems based on psychotherapeutic techniques such as psychiatric talking cures and cognitive-behavioural therapy [6,7,8]. As noted earlier, these techniques aim to integrate traumatic memory into personal life history through talking [51,52]. My research participants also frequently cited the word ‘talking’ (*kuganira*) as an important part of their healing practice. However, they did not talk about traumatic memories for cognitive transformation; rather, they talked about religious and everyday-life issues as a means of social reconnection. Systematic reviews of MHPSS interventions in war-affected populations show that psychiatric and bio-psychological talking practices have very limited effectiveness [7,8]. These limits may be associated with re-traumatisation due to talking about traumatic memories [11,60,61]. Even the local practice of talking about traumatic memories can have negative impacts on mental health and wellbeing. For example, recent evaluations of reconciliation ceremonies in Sierra Leone report that talking about war experiences improved social capital and networks through increasing community participation and reconciliation but also worsened individual mental health as it requires confrontation of traumatic memories [60,61]. The authors note that the social gains are offset by worsened mental health [61]. My findings suggest one possibility to resolve this offset problem. Namely, talking is useful for social reconnection and reconciliation but it is not necessary to touch on traumatic memories and wounds. My findings additionally suggest that the great emphasis on ‘talking’ about trauma can, in effect, isolate sufferers from local communities which ‘practice’ mutual support and ‘living together’. Also, the heavy reliance on the support group can result in lost opportunities to gain social support from communities. This may be another reason why some MHPSS programmes do not fulfil expected outcomes.

### 5.3. Filling the Gap between Mental Health and Psychosocial Support and the Local Community

The findings suggest that isolated sufferers can be (re)integrated through sharing suffering narratives and sharing life with others in the community. The cases of Dieudonné and Muhoza show that ‘talking for mutual understanding’, ‘sharing everyday life’ and ‘helping each other’ can bridge the narrative gaps between MHPSS recipients (mostly Tutsi genocide survivors) and non-recipients (mostly Hutu massacre victims in the Musanze context); this also leads to reconciliation and ethnic integration.

Burnet [62] observes that reconciliation between Tutsi genocide survivors and Hutu massacre victims in Rwanda can happen through sharing verbal narratives of atrocities, exchanging non-verbal signs (e.g., gifts) to acknowledge the other’s suffering and show solidarity and resolving problems step by step in organic communities in which both parties live. My data support her findings and, further, advocate the importance of ‘living together’—i.e., ‘sharing everyday life’ and ‘helping each other’—in the geographical community. In many MHPSS reconciliation programmes in Rwanda, community members are invited as ‘Tutsi victims’ or ‘Hutu perpetrators’ and conversations on traumatic memories are facilitated. In such programmes, ethnic identity and classification as victim or perpetrator tend to be emphasised and other identity aspects neglected. However, in reference to my findings, sharing suffering narratives can take place as part of sharing everyday life, such as working, studying and living together. Kelman [63] suggests a view of ‘reconciliation as identity change’, where both parties stop putting the other’s negation at the centre of their own identity and develop a common, transcendent identity. In the light of his thesis, the everyday setting allows people to live diverse aspects of identity and to perceive aspects other than ‘enemy’ more easily. This can be a window to finding and developing a shared identity between both parties, represented by Dieudonné and Muhoza’s insight, “we were the same”. This study added empirical data to Kelman’s thesis and, further, suggested the significant role of shared practices in an everyday setting to develop a common identity.

### 5.4. Future Collaboration of Mental Health and Psychosocial Support with Local Resilience

Finally, I discuss the ways in which MHPSS could collaborate with local resilience in war-affected settings in the future, in terms of filling the gaps in healing processes as well as facilitating reconciliation. In the light of these findings, this paper supports the psychosocialist theses that MHPSS should be built on local resilience, healing processes and resources, rather than bringing foreign, top-down theories and practices for healing [24,64]. Wessells [24] advocates that bottom-up approaches that build on community assets and resources are sustainable and stimulate collaboration between different sectors, whereas top-down approaches frequently result in low use of formal services and a misalignment of the formal and non-formal systems. This paper additionally notes that when top-down support focuses on one part of the community (those who share the same background) for many years, it can contribute to increasing gaps in socio-economic status and resilience processes within that community, which can even lead to ethnic division.

To mitigate the division, assistance built on local resilience processes, which includes different community members, would be important. This article suggests that better collaboration between MHPSS and local resilience could respond to social aspects of mental health and wellbeing in everyday settings. Some projects have already provided assistances to promote social reconnection and mutual support and achieved successful results [24,65]. However, based on my findings, I emphasise the importance of assisting the process of reconnection and mutual support in everyday settings and in social systems that are already in place within geographical communities. The geographical community has human diversity and attempts to function as one ecological organisation through mutual support amongst different members; different social groups—e.g., faith-based groups and mutual-saving groups, neighbourhood, and kinship groups—play crucial roles in recovery. MHPSS which inclusively supported these social groups in the whole geographical community would allow members to preserve the existing reciprocity and recover the collective life as they are and as they wish to be. Through such support, the community’s healing practices, such as *umusabane* (social party for sharing life), *umuganda* (collective work for helping vulnerable members), and *umubugizi* (mediation of reconciliation), could be recovered with their own initiatives.

MHPSS programmes most frequently select a certain social group, such as victims, women, youths and children, as the target population. In particular in Rwanda, many programmes target genocide survivors. However, drawing from my findings, as well as Kelman’s theory [63], if aid organisations select and intensively support a certain group, it can result in reinforcement of victim and ethnic identities and the healing and reconciliation process can even be interrupted. Reconciliation by conversation between Tutsi genocide survivors and Hutu perpetrators is important. However, to facilitate the ‘reconciliation as identity change’ [63], the support programmes also need to shed light on identity aspects other than ethnicity and roles other than victim and perpetrator; they can then promote mutual help amongst different people within the geographical community. By doing so, as shown by the experience of Dieudonné and Muhoza, each party can go beyond the dichotomic labels of victim and perpetrator and begin to perceive the other as ‘human’.

## Figures and Tables

**Table 1:** Characteristics of research participants.

	Number (Total 40)
Gender	
Female	24
Male	16
Age (range from 22 to 84 years)	
20–29 years	8
30–39 years	17
40 years and over	15
Occupation	
Subsistence farmers	14
Small business owners	5
Non-governmental organisation officers	4
House agents	3
Security guards	3
Students	3
Others (schoolteachers, government officers, cooks, bike riders, tailors, masons)	8

**Table 2:** Interview topic guides.

Main Questions
Q1. Can you tell me your experience during wartime and how you have survived until today? Q2. Can you tell me your experience of how other people helped you with the reconstruction of your life or recovery of your heart? Q3. Can you tell me your experience of how your community/group (e.g., church-based group, mutual-saving group) helped you with the reconstruction of your life or recovery of your heart?

**Table 3:** Locally perceived development of mental health impact of massacres.

*Ibikomere* (Wounded Feelings)	*Ihungabana* (Mental Disturbances)	*Ihahamuka* (Trauma)	*Kurwara mu mutwe* (Illness of the Head)
Emotional problems; most commonly feelings of social isolation and grief—i.e., isolation, loneliness, and helplessness. Sadness, deep sorrow, depression, despair, anxiety/worrying, fear, mistrust are also reported as *ibikomere*.	Behavioural problems; symptoms include social withdrawal, crying all the time, violent behaviour, wrong responses in conversation.	Bio-psychological problems; the word invented as a translation of Western trauma, meaning ‘breathless with frequent fear’. Except for a few who had been trained on trauma, participants did not use it.	Abnormal behaviour; symptoms include social withdrawal, mutism, agitation, hallucinations and nightmares.
Low←	The degree of social isolation and facilitated memories and thoughts on the past		→High

**Table 4:** Healing processes of local communities in Musanze and mental health and psychosocial support (MHPSS).

	Local Communities in Musanze	MHPSS
Conceptualisation of mental health problems	*Ibikomere*: social isolation and grief	*Ihahamuka*; bio-psychological trauma, ‘breathless with frequent fear’ [47,48]
Healing process	‘Living’ together: social reconnection and mutual support in everyday life	Healing happens with multi-layered support, including health, financial, social, educational, livelihood, and legal aid [38,44,45,46]
Healing practices	*Gusura na kuganira* (visiting and talking to each other), *gusangira* (sharing), *gufashanya* (helping each other), *kwiyunga* (reconciliation), *gusenga* (communal prayer) in diverse social groups (i.e., church-based, mutual-saving, kinship and neighbourhood groups)	Talking about traumatic experience and trauma-related problems in the support group
The role of ‘talking’ in the healing process	Talking for reconnection and sharing life: talking about the Bible in church-based groups and talking about the everyday-life problems in mutual-saving groups	Talking for cognitive transformation of traumatic memories and their integration into life history [51,52]
